# Cooling Blood and Detoxicating Formula Treats Psoriasis Through RHCG-Related Mechanisms

**DOI:** 10.1155/ijog/5132158

**Published:** 2025-07-09

**Authors:** Qian Zhang, Juan Huang, Cheng-cheng Feng, Yuan-jie Liu, Ning Yang, Xi Zou, Chen Ji, Shun Guo, Hui Shen

**Affiliations:** ^1^Department of Dermatology, Zhangjiagang TCM Hospital Affiliated to Nanjing University of Chinese Medicine, Zhangjiagang, Jiangsu, China; ^2^Department of Oncology, Affiliated Hospital of Nanjing University of Chinese Medicine, Nanjing, Jiangsu, China; ^3^Department of Acupuncture, Seventh People's Hospital Affiliated to Shanghai University of Traditional Chinese Medicine, Pudong, Shanghai, China; ^4^Department of Dermatology, Affiliated Hospital of Nanjing University of Chinese Medicine, Nanjing, Jiangsu, China

**Keywords:** bioinformatics, cooling blood and detoxicating formula, keratinocytes, psoriasis, RHCG

## Abstract

This study explores the therapeutic potential of the cooling blood and detoxicating formula (CBDF) in the treatment of psoriasis, emphasizing its anti-inflammatory properties and its interaction with RHCG-related mechanisms. Psoriasis, a complex skin disorder characterized by abnormal keratinocyte proliferation and immune system dysregulation, remains challenging to treat effectively. Utilizing advanced techniques including network pharmacology, single-cell RNA sequencing (scRNA-seq), and spatial transcriptomics, this research identifies key active components in CBDF—quercetin and kaempferol—that influence critical inflammatory pathways. In experimental models, CBDF significantly alleviates psoriasis symptoms, reducing keratinocyte differentiation abnormalities and dendritic cell (DC) activation. Molecular docking studies demonstrate strong interactions between RHCG and the active ingredients in CBDF. These findings suggest that CBDF exerts its effects through a multifaceted approach, with RHCG identified as a pivotal target. While the results are promising, further clinical validation and mechanistic research are needed. This study underscores the potential of CBDF as a treatment for psoriasis, blending traditional medicine with modern molecular insights.

## 1. Introduction

Psoriasis is a chronic, immune-mediated skin disease affecting millions worldwide, with a strong genetic predisposition and a tendency to worsen during winter months [1]. Although current therapeutic strategies—including lifestyle modifications, topical treatments, systemic agents targeting inflammation, and plaque formation, as well as phototherapy and laser therapy—have significantly improved disease management [2], achieving sustained remission remains challenging. Factors such as treatment resistance, variability in patient responses, frequent disease recurrence, and adverse effects associated with long-term therapy limit the overall effectiveness of available options [3].

Traditional Chinese medicine (TCM) offers a complementary approach, providing individualized treatment plans based on syndrome differentiation. By addressing underlying imbalances and promoting holistic health, TCM therapies aim to achieve long-term remission and improve patients' quality of life [4, 5].

In TCM, psoriasis—historically referred to as “Baibi”—is classified according to blood-related syndromes, including blood heat, blood stasis, and blood dryness [6, 7]. Among these, blood heat syndrome is the most prevalent, accounting for approximately 53.8% of psoriasis vulgaris cases, and is considered central to disease progression [8]. To specifically target this syndrome, the Dermatology Department of Beijing Hospital of TCM developed the Cooling Blood and Detoxicating Formula (CBDF), which has demonstrated notable clinical efficacy [9]. Several studies have examined CBDF's regulatory effects on immune function and inflammatory mediators, and recent evidence suggests that CBDF may also modulate the metabolic phenotype of dendritic cells (DCs) [10, 11]. However, further research is needed to elucidate the active components and precise mechanisms underlying CBDF's therapeutic effects. Our previous investigations have revealed that Rh family C glycoprotein (RHCG) may serve as a critical factor in the pathogenesis of psoriasis [12], particularly by influencing the abnormal differentiation of keratinocytes and the activation of DCs [13]. This novel finding provides a potential mechanistic link for further exploration of CBDF's effects. Therefore, exploring the connection between CBDF's anti-inflammatory effects and RHCG-related signaling pathways may offer new insights into the mechanisms of CBDF efficacy.

In this study, we employed network pharmacology to identify the active ingredients and potential molecular targets of CBDF. Furthermore, we integrated single-cell RNA sequencing (scRNA-seq) and spatial transcriptomics sequencing (ST-seq) technologies to investigate how CBDF modulates RHCG-associated signaling pathways in the treatment of psoriasis.

## 2. Materials and Methods

### 2.1. Materials and Reagent

CBDF was obtained from the Pharmacy Department of Zhangjiagang Traditional Chinese Medicine Hospital. The detailed composition of CBDF is provided in Table S1. All additional materials and reagents utilized in this study are listed in Table S2.

### 2.2. Preparation of CBDF Decoction Mixture

To prepare the CBDF extract, 154 g of the CBDF mixture was soaked in tenfold volume of purified water (*w*/*v*) for 30 min at room temperature. The mixture was then subjected to reflux extraction for 120 min. Following extraction, the aqueous solution was filtered, and the solid residue was collected for a second reflux extraction using eightfold volume of purified water (*w*/*v*) for an additional 60 min. The filtrates from both extractions were combined and concentrated under reduced pressure at 40°C and approximately 100 mbar using a vacuum evaporator (Rotavapor R-200, Buchi, Switzerland). The resulting concentrated solution was adjusted to a medium-dose extract with a final concentration of 2 g/mL. To obtain the high-dose preparation, the medium-dose extract was further concentrated to 4 g/mL, whereas the low-dose preparation was achieved by diluting the medium-dose extract to 1 g/mL with purified water. The dosing regimen for pharmacological studies in mice was calculated based on body surface area normalization to ensure consistency and clinical relevance. Specifically, the human equivalent dose (HED) was converted to the animal dose using the following formula: Animal dose (mg/kg) = HED (mg/kg) × (human Km/mouse Km). According to the FDA-recommended Km values, a factor of 37 was used for humans and 3 for mice. This approach ensured dosing consistency and clinical relevance for subsequent pharmacological evaluations [14].

### 2.3. Psoriasis-Like Animal's Model Construction and CBDF Administration

A total of 42 specific pathogen-free (SPF) female C57BL/6J (B6) mice, aged 6–8 weeks and weighing approximately 18–22 g, were obtained from Henan Scribes Biotechnology Co., Ltd. (License Number: SCXK [YU] 2020-0005). The mice were housed under controlled conditions (22 ± 2°C, 55 ± 10% humidity) with a 12-h light/dark cycle, and were allowed free access to standard laboratory chow and water. All experimental procedures were approved by the Animal Care Committee of Zhangjiagang Traditional Chinese Medicine Hospital (Zhangjiagang, China; approval number: KY-2023-11-09-01) and conducted in accordance with the Animal Care and Use Guidelines of the National Institutes of Health.

A total of 42 specific pathogen-free (SPF) female C57BL/6J (B6) mice (6–8 weeks old, weighing 18–22 g) were randomly assigned into seven groups (*n* = 6 per group): (1) blank control group, (2) IMQ model group, (3) methotrexate (MTX) group, (4) secukinumab group, (5) high-dose CBDF group, (6) medium-dose CBDF group, and (7) low-dose CBDF group.

Prior to modeling, the dorsal hair of each mouse was carefully shaved to expose an area of approximately 2 cm × 3 cm. Psoriasis-like skin inflammation was induced in the model and treatment groups by daily topical application of 5% imiquimod (IMQ) cream (62.5 mg) to the shaved area for 5 consecutive days.

The MTX group received methotrexate at a dose of 1 mg/kg/day via oral gavage for 7 days. The secukinumab group was administered secukinumab intraperitoneally at a dose of 10 mg/kg/day for 5 days. Mice in the CBDF treatment groups were orally administered 0.2 mL of CBDF decoction daily at high, medium, or low doses, respectively. The blank control group received an equivalent amount of Vaseline topically and distilled water orally.

Clinical signs were monitored daily, and the severity of skin lesions was assessed using a modified Psoriasis Area and Severity Index (PASI) scoring system (severity score), based on erythema, scaling, and thickness.

### 2.4. Scoring Severity of Skin Inflammation

The severity of skin lesions was evaluated daily using a modified scoring system based on three clinical parameters: erythema, scaling, and thickness. Each parameter was scored independently on a scale from 0 to 4 (0 = *none*; 1 = *slight*; 2 = *moderate*; 3 = *marked*; and 4 = *very marked*). The total severity score was calculated as the sum of these three individual scores, with a maximum possible score of 12. Area involvement was not considered in this scoring system.

Histological evaluation was performed to assess epidermal hyperplasia and inflammatory cell infiltration. Infiltration scores were assigned based on hematoxylin–eosin (HE)–stained skin sections according to the following criteria: 0 = *no infiltration*; 1 = *mild infiltration*; 2 = *moderate infiltration*; 3 = *marked infiltration*; and 4 = *extensive infiltration throughout the dermis*. All scoring was conducted in a blinded manner by two independent investigators.

### 2.5. Identification of Active Ingredients and Potential Targets

A comprehensive search for the 10 Chinese herbs constituting CBDF—Imperatae Rhizoma, Dictamni Cortex, Radix Paeoniae Rubra, Rehmanniae Radix Praeparata, *Sophora japonica* L., Lonicerae Japonicae Flos, Sophorae Flavescentis Radix, Bistortae Rhizoma, Smilacis Glabrae Rhizoma, and *Lithospermum erythrorhizon*—was conducted using the Traditional Chinese Medicine Database and Analysis Platform (TCMSP) [15]. Active compounds were selected based on criteria of oral bioavailability (OB) ≥ 30% and drug‐likeness (DL) ≥ 0.18, ensuring the inclusion of compounds with favorable pharmacokinetic properties and potential for oral administration [16].

Following the identification of active compounds, all associated potential targets were retrieved in TCMSP (Version 2.3). To ensure consistency in target annotation, target names were standardized to their corresponding gene symbols using the STRING (Version 12.0) database [17]. Duplicates were subsequently removed to eliminate redundancy, and nonhuman targets were excluded to focus solely on targets relevant to human biology. This systematic approach allowed for the definitive identification of compound-associated targets, providing a foundation for subsequent network construction and mechanistic analysis.

### 2.6. Enrichment Analysis

The CBDF's potential targets were imported into the clusterProfiler package [18] to perform GO (Gene Ontology) analysis to determine BP (biological process), CC (cellular component), and MF (molecular function) term enrichment and perform KEGG (Kyoto Encyclopedia of Genes and Genomes) enrichment. The ggplot2 package [19] was conducted to visualize the results of the enrichment analysis.

### 2.7. RHCG-Related Gene Identification

The RHCG-related gene data were reviewed in the “gene and disease bank” module from the citexs platform.

### 2.8. Molecular Docking

Molecular docking was conducted using the CB-Dock2 online tool, a blind docking platform based on AutoDock Vina [20]. Default parameters were applied throughout the docking process to maintain consistency and reproducibility. The molecular structures of the active ingredients in CBDF (e.g., quercetin, kaempferol, luteolin, wogonin, and formononetin) were retrieved from the PubChem database, a comprehensive repository of chemical information. The three-dimensional structure of the RHCG protein was obtained from the RCSB Protein Data Bank [21], a widely recognized resource for structural biology. Prior to docking, both the active compounds and the RHCG protein were prepared through protonation and energy minimization to optimize their structural states. This molecular docking approach enabled the evaluation of binding interactions between the CBDF active ingredients and the RHCG protein, offering insights into their structural compatibility and binding affinities.

### 2.9. scRNA-Seq Data Source and Analysis

scRNA-seq data were obtained from the Gene Expression Omnibus (GEO) database, comprising 11 samples of peripheral normal skin and 15 samples of psoriatic skin from psoriasis patients (GSE173706 and GSE206147) [22, 23]. To eliminate batch effects, all samples were integrated using the Harmony package [24], based on the Top 50 principal component analysis (PCA) components. Following harmonization, *k*-nearest neighbors (k-NNs) were computed, and a shared nearest neighbor (SNN) graph was subsequently constructed. Clustering was performed by optimizing the modularity function to facilitate the identification of distinct cell populations. The resulting clusters were visualized using uniform manifold approximation and projection (UMAP) for dimensionality reduction. For cell type annotation, known marker genes were retrieved from the DISCO database [25] and manually compared with specific genes identified in this study to infer cell types. Additionally, visualization of cell types and gene or gene set expression was carried out using the Scillus package [26].

### 2.10. ST Data Source and Analysis

ST data were retrieved and downloaded from the GEO database with the access number GSE225475 [27]. A total of 4 psoriatic skin samples were included in this study.

For ST data processing, we used the “SPATA” package [28] to calculate and visualize gene/gene set expression. For unbiased clustering and cell type identification, we utilized the clustering and deconvolution results provided by the CROST platform [29].

### 2.11. HE Staining

Skin tissue samples from C57BL/6J mice were fixed in 4% paraformaldehyde (PFA) at 4°C for 24 h, followed by dehydration through a graded ethanol series, clearing in xylene, and embedding in paraffin. Paraffin blocks were sectioned into 4-*μ*m-thick slices, mounted on glass slides, and dried overnight at 37°C. For staining, tissue sections were deparaffinized in xylene and rehydrated through descending ethanol gradients. Sections were stained with hematoxylin for 5 min, rinsed under running tap water, and differentiated with 1% acid alcohol for 30 s, followed by bluing in ammonia water. Subsequently, sections were counterstained with eosin for 2 min, dehydrated through ascending ethanol gradients, cleared in xylene, and mounted using Distrene Plasticizer Xylene (DPX) mounting medium. Stained sections were examined under a light microscope (Olympus BX53, Olympus Corporation, Tokyo, Japan) to assess tissue morphology and pathological features.

### 2.12. Tissue Section Immunofluorescence Staining

For immunofluorescence staining, sections were deparaffinized with xylene, rehydrated through a descending ethanol series, and subjected to antigen retrieval by heating in citrate buffer (pH 6.0) for 20 min. After cooling to room temperature, sections were blocked with 5% bovine serum albumin (BSA) for 1 hour and then incubated overnight at 4°C with primary antibodies (details listed in Table S2). After washing with PBS, sections were incubated with appropriate fluorescently labeled secondary antibodies for 1 h at room temperature in the dark. Nuclei were counterstained with DAPI for 5 min. Finally, sections were mounted with antifade mounting medium.

Fluorescence images were acquired using an Olympus fluorescence microscope (Olympus BX53, Olympus Corporation, Tokyo, Japan) under identical exposure settings across all groups. Quantification of fluorescence intensity in psoriasis lesion areas was performed using ImageJ software (Version 2.0.0).

## 3. Results

### 3.1. The Therapeutic Mechanism of CBDF Is Related to Anti-Inflammatory Effects

**Figure 1:**
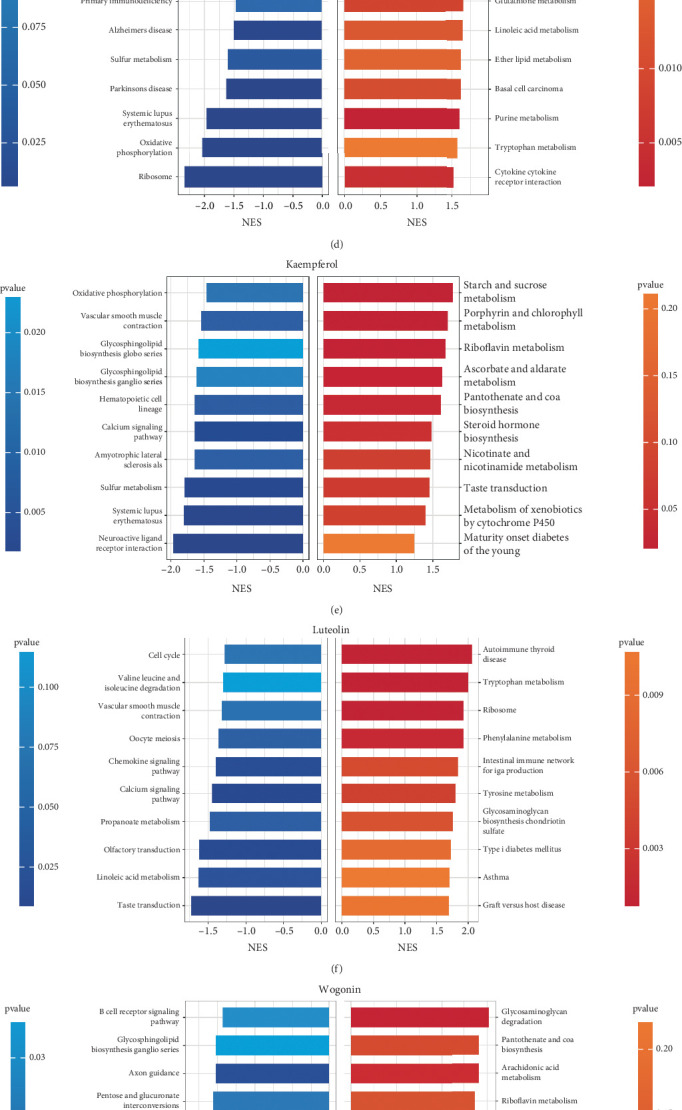
Compound–target network construction, active compound identification, and pathway analysis of CBDF in psoriasis treatment. (a) Psoriasis-related pathogenic genes identified using the Citexs web tool, demonstrating a highly interconnected network involving key inflammation-related genes such as TNF, CD4, IL-10, and IL-4, all central to the inflammatory cascade in psoriasis. (b) Compound–target network analysis of CBDF constructed using the TCMSP database and visualized in Cytoscape 3.8.2. The network highlights interactions between bioactive compounds and potential target genes. (c) Ranking of bioactive components by degree centrality. Quercetin exhibited the highest degree value (143), followed by kaempferol (60), luteolin (55), wogonin (45), and formononetin (37), indicating their pivotal roles in the therapeutic effects of CBDF. (d–h) Pathway enrichment analysis of the top five active ingredients: quercetin, kaempferol, luteolin, wogonin, and formononetin. Results revealed their suppressive effects on oxidative phosphorylation and their significant association with inflammation regulation. The NES (normalized enrichment score) and *p* value indicate the enrichment and significance of specific pathways. (i) Bubble plot illustrating the regulatory effects of the five major active ingredients on psoriasis-related inflammatory mediators. The size and color of the bubbles represent the magnitude and direction of expression changes, respectively, confirming the anti-inflammatory properties of CBDF.

We first identified psoriasis-related pathogenic genes using the Citexs web tool (https://www.citexs.com). As shown in Figure 1a, the development of psoriasis involves numerous inflammation-related genes, including *TNF* (encoding tumor necrosis factor), *CD4* (encoding the CD4 molecule), *IL-10* (encoding Interleukin-10), and *IL-4* (encoding Interleukin-4). Psoriasis is well known to be intricately linked to inflammation, characterized by a complex interplay between immune system dysregulation and epidermal hyperproliferation, leading to the characteristic skin lesions observed in this chronic immune-mediated disorder [30–32]. Moreover, the inflammatory cascade in psoriasis involves a wide array of cytokines and chemokines, establishing a proinflammatory microenvironment that perpetuates disease progression and exacerbates clinical symptoms [33, 34]. Previous studies have demonstrated that CBDF can modulate immune function and inflammatory response [11]. Therefore, we focused our investigation on the anti-inflammatory potential of CBDF.

Potential targets of CBDF were identified and mapped using the STRING database and subsequently standardized to official gene symbols. The active components and potential targets were then imported into Cytoscape 3.8.2 for network analysis, resulting in a compound–target interaction map (Figure 1b). Based on degree centrality rankings, quercetin, kaempferol, luteolin, wogonin, and formononetin were identified as the principal bioactive components (Figure 1c). Notably, quercetin, present in six herbs and possessing the highest degree value (143), appeared to be the most important active ingredient. Numerous studies have documented the anti-inflammatory and immunomodulatory effects of quercetin [35, 36].

To further elucidate the potential efficacy of the main active components in CBDF, we queried the ITCM database for their associated biological signaling pathways. As shown in Figures 1d, 1e, 1f, 1g, and 1h, quercetin, kaempferol, and formononetin were found to inhibit oxidative phosphorylation. While there is currently no direct evidence supporting the regulation of oxidative phosphorylation by these compounds in the context of psoriasis, their anti-inflammatory activities and therapeutic potential in psoriasis have been well documented [37–40]. An intricate relationship exists between oxidative phosphorylation and inflammation, wherein disturbances in oxidative phosphorylation have been implicated in modulating inflammatory responses, and conversely, inflammatory stimuli can impact mitochondrial function [41, 42]. Using network pharmacology analysis, we predicted the regulatory effects of CBDF components on key psoriasis-associated molecules. As shown in Figure 1i, the majority of molecules, were downregulated (blue dots). Notably, quercetin and formononetin upregulated TNF-*α* (red dots), likely reflecting a transient, context-dependent modulation within a broader regulatory network. This upregulation may involve immune cell recruitment or feedback mechanisms that ultimately contribute to inflammation resolution, supporting CBDF's predominantly anti-inflammatory profile.

### 3.2. Analysis of the Potential Target Genes of CBDF for the Treatment of Psoriasis

**Figure 2:**
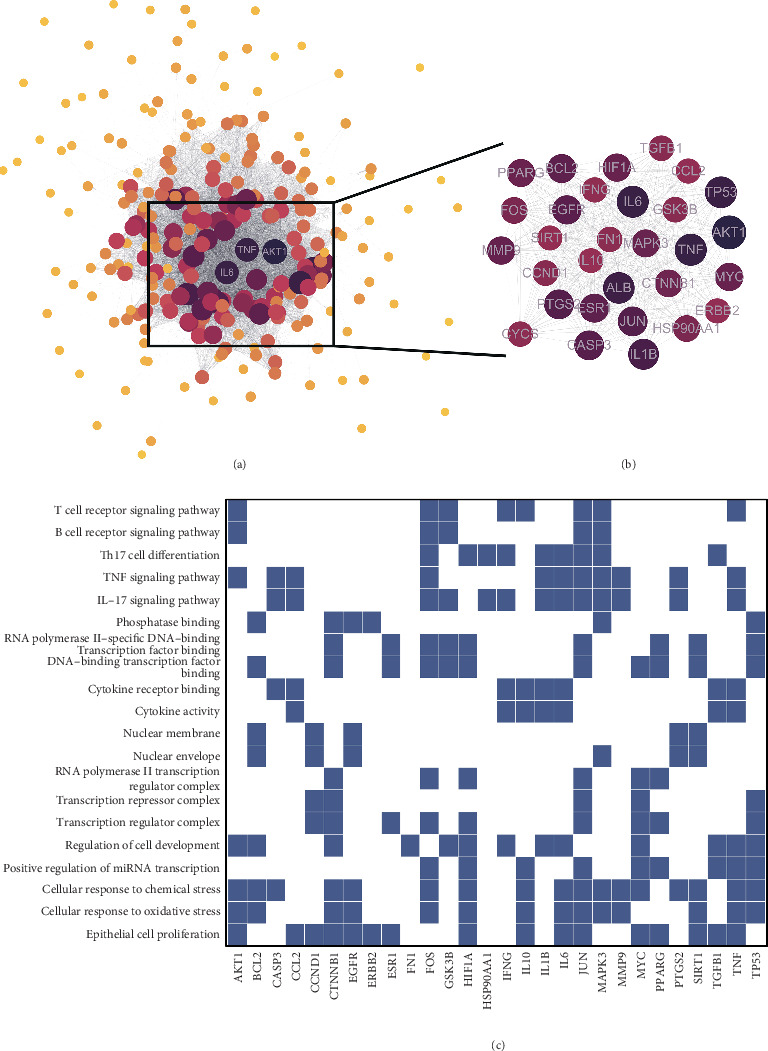
PPI network construction and pathway enrichment analysis of CBDF target genes for psoriasis treatment. (a) Protein–protein interaction (PPI) network depicting the interactions among potential target genes of CBDF in psoriasis treatment. Nodes represent genes, and their size reflects degree centrality, with larger nodes indicating greater connectivity. (b) Highly interconnected subcluster identified using the MCODE plugin in Cytoscape. The subcluster includes 30 hub genes with the highest scores, such as TNF, IL-6, IL-1B, AKT1, and ALB, which are pivotal targets in the therapeutic mechanisms of CBDF. (c) Heatmap summarizing GO and KEGG pathway enrichment analyses for the identified subcluster. The Top 20 pathways are shown, including immune-related pathways (e.g., T cell receptor signaling, B cell receptor signaling, and IL-17 signaling pathways), nuclear transcription, and cellular stress response mechanisms. Blue squares indicate significant associations between hub genes and biological processes/pathways.

A protein–protein interaction (PPI) network was meticulously constructed with CBDF's potential targets by STRING database to elucidate the complex interaction landscape among the target genes under investigation (Figure 2a). The targets were further analyzed using the MCODE plugin in Cytoscape, screening for highly interconnected subclusters with scores greater than 100 (Figure 2b). Through this analysis, 30 hub genes were identified. The top five targets in this network were *TNF*, *IL-6* (encoding Interleukin-6), *IL-1B* (encoding Interleukin-1*β*), *AKT1* (encoding AKT Serine/Threonine Kinase 1), and *ALB* (encoding albumin). Previous studies have shown significant decreases in IL-2 receptor, IL-6, and TNF-*α* levels after acitretin capsules and CBDF treatment, in partial agreement with our predictions here [43].

Subsequently, GO and KEGG enrichment analyses were performed to explore the biological functions and metabolic pathways associated with the key subcluster. The heat map derived from the enrichment analysis (Figure 2c) illustrates the potential impact of CBDF on psoriasis. Among the 2892 enriched pathways, we focused on 20 pathways, selecting the top five under each functional category. These pathways are closely associated with a range of pathophysiological processes, particularly those related to immune system regulation, nuclear transcription, and proliferation-related signaling mechanisms. It has been shown that CBDF attenuates psoriatic pathologies by modulating metabolic reprogramming pathways through an AMPK-dependent signaling axis, thereby suppressing DC maturation and mitigating disease progression [44]. Our results further support a role for CBDF in immune and inflammatory regulation.

### 3.3. scRNA-Seq Reveals That the Therapeutic Mechanism of CBDF Involves Multiple Cell Types, Especially Keratinocytes

**Figure 3:**
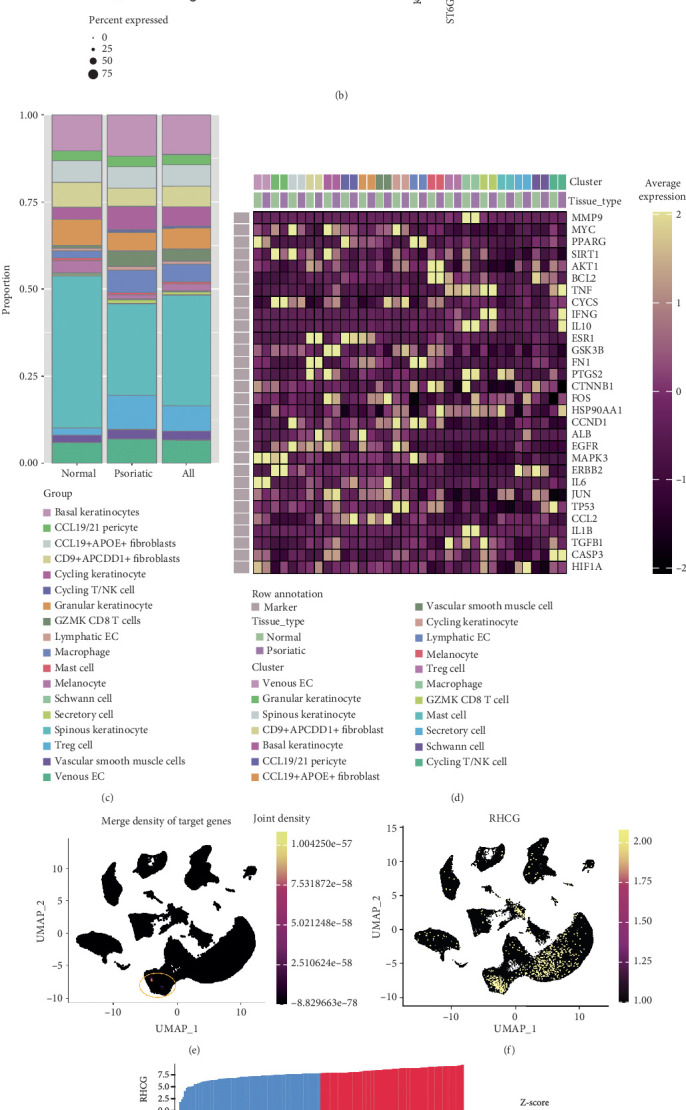
scRNA-seq analysis reveals CBDF's therapeutic mechanism through multiple cell types, with a focus on keratinocytes. (a) UMAP visualization of scRNA-seq data (GSE173706 and GSE206147) from psoriatic and normal skin tissues. A total of 99838 cells were classified into 18 distinct cell lineages based on canonical cell markers. (b) Dot plot illustrating the expression profiles of key marker genes across identified cell types. Dot size represents the percentage of cells expressing each marker, and color intensity indicates the average expression level. (c) Proportional comparison of cell type distribution between normal and psoriatic tissues. Notable increases in cycling keratinocyte, GZMK CD8 T cell, macrophage, and Treg cell populations were observed in psoriatic samples. (d) Heatmap showing the single-cell expression of 30 hub genes across different cell types, highlighting significant expression differences between normal and psoriatic tissues. (e) Joint density estimation of the 30 hub genes demonstrates their combined expression predominantly in basal keratinocytes, suggesting these cells as primary CBDF targets. (f) UMAP visualization of RHCG expression, indicating its prominent role in basal keratinocytes. (g) Correlation heat map of RHCG with the 30 hub genes based on scRNA-seq and bulk-seq data, showing significant relationships, particularly with genes involved in inflammation and keratinocyte differentiation.

scRNA-seq technology allows researchers to investigate gene expression with high precision, enabling the dissection of complex gene regulatory networks within individual cells and revealing subtle differences across cellular compartments. In this study, we analyzed scRNA-seq data from the GSE173706 and GSE206147 datasets, comprising 15 psoriatic and 11 normal skin tissue samples. After performing quality control and correcting for batch effects, we retained 99,838 cells, which were subsequently annotated into distinct cell lineages based on established markers. These included vascular smooth muscle cells, CD9^+^APCDD1^+^ fibroblasts, CCL19/21 pericytes, CCL19^+^APOE^+^ fibroblasts, melanocytes, lymphatic endothelial cells, venous endothelial cells, Schwann cells, mast cells, T regulatory (Treg) cells, macrophages, secretory cells, granular keratinocytes, GZMK^+^ CD8 T cells, cycling keratinocytes, cycling T/NK cells, spinous keratinocytes, and basal keratinocytes (Figure 3a,b). The distribution of these cell lineages varied markedly between psoriatic and normal tissues (Figure 3c), with an increased proportion of cycling keratinocytes, GZMK^+^ CD8 T cells, macrophages, and Treg cells observed in psoriasis samples.

We next examined the expression profiles of 30 hub genes at the single-cell level and found that these genes were expressed across multiple cell types, displaying significant differences between normal and psoriatic tissues (Figure 3d. These findings suggest that the therapeutic effects of CBDF involve a range of CCs. Furthermore, a joint density estimation based on the expression of the 30 hub genes revealed that their combined expression was most prominent in basal keratinocytes (Figure 3e), indicating that keratinocytes may represent a major target of CBDF.

Our research group has previously explored the role of RHCG in the pathogenesis of psoriasis, demonstrating its critical involvement in keratinocyte dysregulation and inflammatory responses. Given that CBDF exhibits potent therapeutic effects on inflammatory phenotypes, we hypothesized that CBDF may modulate RHCG expression. Supporting this hypothesis, both scRNA-seq and bulk RNA-seq data revealed a clear correlation between RHCG and CBDF target gene expression (Figure 3f,g).

### 3.4. ST Analysis Reveals That the Therapeutic Mechanism of CBDF Implicated Specific Spatial Domains

**Figure 4:**
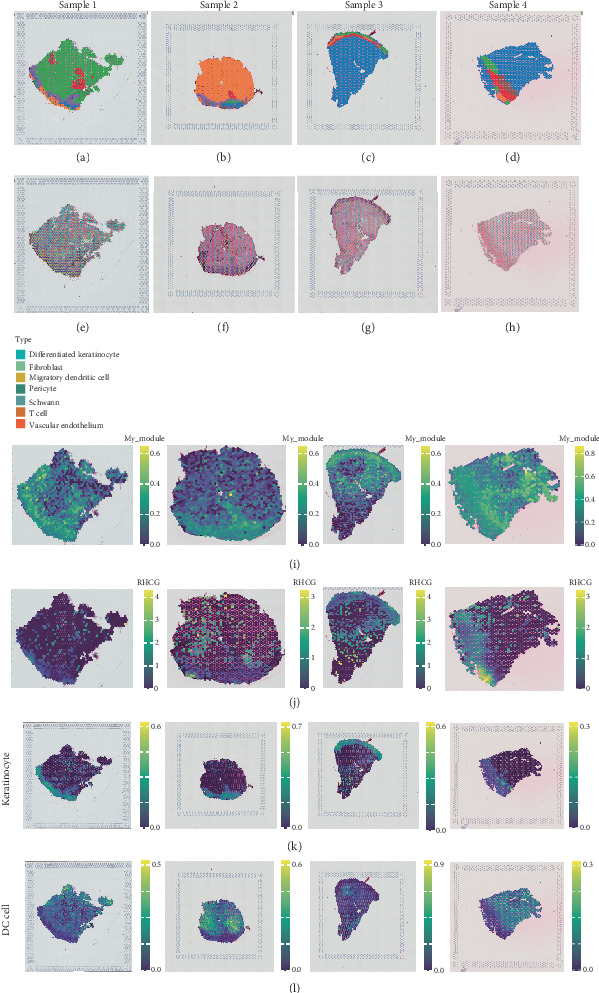
Spatial transcriptomics analysis reveals CBDF's therapeutic mechanism in specific spatial domains. (a–d) Unbiased clustering of spatial transcriptomics data from Samples 1–4. Spots were grouped into distinct clusters based on spatial features and gene expression profiles. (e–h) Cell type annotation of clusters using CROST deconvolution data. Identified cell types include differentiated keratinocyte, fibroblast, migratory dendritic cell, pericyte, Schwann, T cell, and vascular endothelium. (i) Spatial expression profiles of 30 hub genes in psoriasis samples. My_module = 30 hub genes exhibited in Figure 2b. (j) Spatial distribution of RHCG expression across samples, demonstrating its presence in specific spatial domains. The spatial gene expression signature is highly similar to RHCG, suggesting a strong correlation between RHCG and CBDF's therapeutic mechanism. (k, l) Overlap between spatial domains of keratinocytes (K) and migratory dendritic cells (DCs) (l) the expression of hub genes, emphasizing their involvement in CBDF's mechanism.

We further explored the relationship between the therapeutic mechanism of CBDF and RHCG within the spatial architecture of psoriatic tissues (Samples 1–4). Using unbiased clustering based on spot features, we classified the spatial transcriptomic spots into distinct clusters (Figures 4a, 4b, 4c, and 4d)). Through deconvolution analysis with CROST, these clusters were annotated to specific cell types, including differentiated keratinocytes, fibroblasts, migratory DCs, pericytes, Schwann cells, T cells, and vascular endothelial cells (Figures 4e, 4f, 4g, and 4h).

Next, we examined the expression profiles of the 30 hub genes across the psoriasis samples. Our findings revealed that these hub genes exhibit a distinct expression signature closely resembling that of RHCG (Figure 4i,j). Notably, there was a significant spatial overlap between the domains expressing these hub genes and the regions enriched for keratinocytes and DCs (Figure 4k,l). Together, these results further support the notion that CBDF's therapeutic mechanism may involve modulation of RHCG-associated cellular states.

### 3.5. Molecular Docking of Representative Active Ingredients of CBDF and RHCG

**Figure 5:**
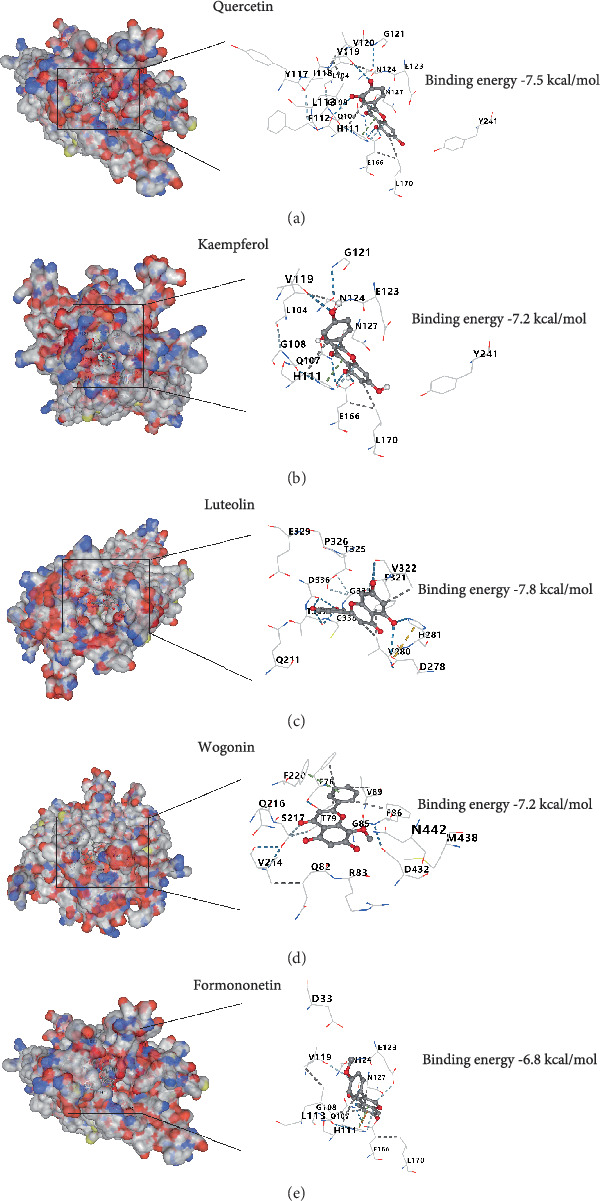
Molecular docking analysis of CBDF's representative active ingredients with RHCG. Docking poses and binding energies of the five primary active components of CBDF with RHCG: (a) quercetin (−7.5 kcal/mol), (b) kaempferol (−7.2 kcal/mol), (c) luteolin (−7.8 kcal/mol), (d) wogonin (−7.2 kcal/mol), and (e) formononetin (−6.8 kcal/mol). Binding interactions are visualized within the RHCG binding pocket, highlighting the spatial alignment and molecular interactions. The low binding energies suggest strong binding affinities, with luteolin exhibiting the most favorable binding energy (−7.8 kcal/mol), indicating its potential role as a key modulator of RHCG activity.

To further validate the representative active components of CBDF based on drug–target interactions, we performed molecular docking analyses between RHCG and five major active ingredients: quercetin, kaempferol, luteolin, wogonin, and formononetin. As shown in Figures 5a, 5b, 5c, 5d, and 5e, all compounds exhibited binding energies below −6 kcal/mol, which is generally considered indicative of moderate to strong binding affinity. Among them, luteolin demonstrated the strongest binding to RHCG, with a binding energy of −7.8 kcal/mol. To confirm these interactions functionally, we conducted in vitro experiments in an M5-induced HaCaT cell model, using maximum nontoxic concentrations of each compound to demonstrate differential RHCG expression (Figure S1A). Western blot analysis revealed that these compounds significantly inhibited RHCG protein expression compared to the M5 model group (Figure S1B). These findings suggest that the active constituents of CBDF may downregulate RHCG expression, potentially contributing to their therapeutic effects. However, whether RHCG functions as a downstream effector or merely reflects secondary changes in response to treatment remains to be determined.

### 3.6. CBDF Downregulated the Levels of RHCG and DC Activation

**Figure 6:**
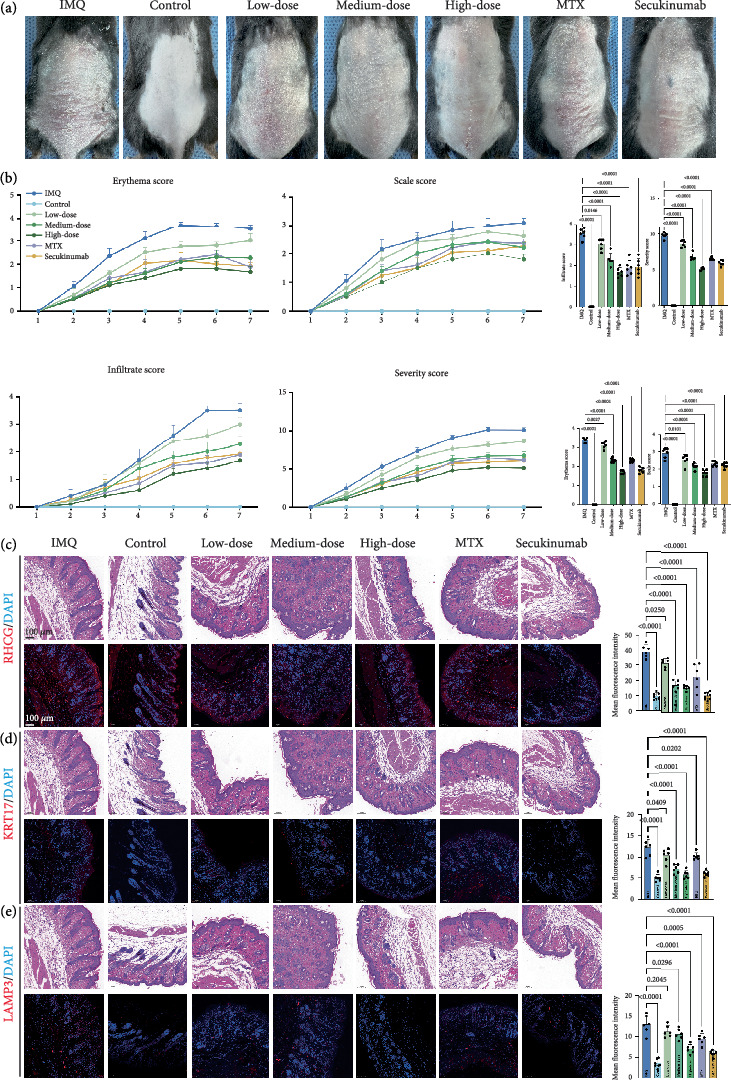
CBDF mitigates psoriatic lesions and regulates RHCG and DC activation in an IMQ-induced psoriasis mouse model. (a) Representative images of dorsal skin from IMQ group, control group, and treatment groups (low-, medium-, and high-dose CBDF, MTX, and secukinumab). CBDF treatment markedly reduced erythema, scaling, and thickening of psoriatic lesions compared to the IMQ group. (b) Erythema, scale, infiltrate, and severity score across different groups. CBDF significantly reduced these scores. (c–e) HE staining and immunofluorescence analysis of dorsal skin sections. HE staining of skin sections from the IMQ, control, low-dose, medium-dose, high-dose, MTX, and secukinumab groups is shown. The IMQ group exhibited typical pathological features of psoriasis, including parakeratosis, hyperkeratosis, and inflammatory infiltration, which were significantly alleviated by CBDF, MTX, and secukinumab. The effect of CBDF was dose-dependent, with the high-dose group showing the most significant improvement. Immunofluorescence staining of RHCG (c), KRT17 (d), and LAMP3 (e) was performed to assess the markers of keratinocyte differentiation and dendritic cell (DC) activation. Their expression was notably reduced following treatment with CBDF (especially in the medium- and high-dose groups), MTX, and secukinumab. Scale bar = 100 *μ*m. Data are presented as mean ± SD (*n* = 6). Statistical significance was assessed using one-way ANOVA followed by Tukey's post hoc test. *p* values are indicated for each comparison.

The regulatory effect of CBDF on psoriasis was further validated through animal experiments. Throughout the study, mice in the control group maintained normal physiological parameters, including stable food and water intake and regular breathing patterns. Following IMQ administration, the model group exhibited pronounced signs of erythema, scaling, and increased skin thickness within 2 days, in stark contrast to the control group. In mice treated with CBDF, a significant amelioration of psoriatic skin symptoms was observed (Figure 6a). Additionally, CBDF treatment resulted in a notable reduction in the severity score compared to the model group (Figure 6b). Histological analysis using HE staining showed that the skin of control mice had a thin epidermis with normally structured cell layers and no pathological changes. In contrast, the model group displayed typical psoriatic pathology, including parakeratosis, hyperkeratosis, thickening of the spinous layer, elongation of epidermal rete ridges, and inflammatory cell infiltration. Importantly, treatment with CBDF, MTX, and secukinumab led to varying degrees of improvement in these pathological features, with more pronounced effects observed in the high- and middle-dose CBDF groups compared to the low-dose group (Figures 6c, 6d, and 6e, top panel).

We assessed the expression of RHCG and KRT17—markers of abnormal keratinocytes differentiation—using immunofluorescence staining. As expected, both RHCG and KRT17 levels were markedly elevated in the model group compared to the control group, whereas treatment with CBDF, MTX, and secukinumab effectively reversed these changes, particularly in the middle- and high-dose CBDF groups. Furthermore, given that keratinocytes often interact with DCs to exacerbate psoriatic inflammation [45, 46], we also examined the expression of LAMP3, a marker of DC activation. LAMP3 expression was significantly upregulated in the model group compared to controls but was notably reduced following treatment with CBDF (especially at middle and high doses), MTX, and secukinumab (Figures 6c, 6d, and 6e, bottom panel).

## 4. Discussion

Despite extensive research efforts, the intricate pathophysiological mechanisms underlying psoriasis remain incompletely understood. This chronic inflammatory skin disease arises from a complex interplay of genetic predisposition, immune dysregulation, environmental triggers, and epidermal hyperproliferation, all of which contribute to the ongoing challenge of fully elucidating its pathogenesis [47, 48]. Central to psoriasis is the dynamic crosstalk between keratinocytes and immune cells [49]. Keratinocytes exhibit abnormal proliferation and differentiation, while immune cells—particularly T cells—migrate into the skin and initiate inflammatory responses [50]. This interaction leads to the secretion of cytokines and chemokines, sustaining a chronic inflammatory cycle and promoting the development of characteristic skin lesions [49]. Moreover, the persistence of tissue-resident memory T cells further complicates the pathobiology of psoriasis, highlighting the need for continued research into its underlying mechanisms [45].

The complex, multitarget, and multicellular mechanisms underlying psoriasis present a unique opportunity for the application of TCM [51]. Given the intricate interplay among diverse cellular populations and signaling pathways in psoriasis, TCM's holistic approach—emphasizing the balance and harmony of qi and blood—may be particularly well-suited to address this complex pathophysiology [52, 53]. By simultaneously targeting multiple aspects of inflammatory and immune responses, and modulating epidermal proliferation and differentiation, TCM offers a complementary therapeutic strategy that holds promise for improving outcomes in patients with psoriasis [54–56].

Analysis of the drug–target network reveals the intricate composition of CBDF, the diversity of its molecular targets, and the extensive array of action pathways involved. The therapeutic efficacy of CBDF appears to arise from the synergistic effects of multiple bioactive components acting in concert. Notably, constituents such as quercetin, kaempferol, and luteolin—members of the flavonoid family—exhibit potent anti-inflammatory properties [57–59]. These compounds inhibit the nuclear factor-*κ*B (NF-*κ*B) signaling pathway, suppressing the transcription of inflammatory genes and thereby reducing the production of cytokines and chemokines, including interleukin-1*β* (IL-1*β*), interleukin-6 (IL-6), and tumor necrosis factor-*α* (TNF-*α*) [60]. Additionally, they scavenge reactive oxygen species (ROS) and reactive nitrogen species (RNS), mitigating oxidative stress and further dampening inflammatory responses [61]. By modulating inflammatory cascades and enhancing antioxidant defenses, quercetin, kaempferol, and luteolin serve as promising natural agents for managing inflammatory disorders, including psoriasis. While CBDF's therapeutic impact extends beyond the effects of its individual components, the anti-inflammatory properties of these flavonoids likely contribute substantially to its antipsoriatic efficacy.

We employed a network-based approach and constructed a CBDF–target interaction network using PPI data of predicted targets and associated proteins. By extracting core targets, we identified 20 key pathways through GO and KEGG enrichment analyses. The results indicated that CBDF treatment for psoriasis is associated with modulation of the immune system, nuclear transcription processes, epithelial cell proliferation, and various other signaling pathways. These findings suggest that CBDF may exert therapeutic effects through multiple biological mechanisms. Psoriasis is characterized by an exaggerated immune response, with proinflammatory cytokines such as TNF-*α*, IL-1, and IL-6 contributing to disease pathogenesis through sustained inflammation [62, 63]. Notably, scRNA-seq and spatial transcriptomics analyses provided clear evidence that CBDF targets multiple cell types, particularly keratinocytes and DCs. The interaction between keratinocytes and DCs is critical in amplifying psoriatic immunopathology [64]. Abnormally proliferating keratinocytes, along with activated DCs, promote antigen presentation and T-cell activation, leading to the secretion of cytokines such as TNF-*α*, IL-12, IL-23, and IL-17. This cytokine storm sustains the inflammatory microenvironment, attracting neutrophils and Tc1 cells, and further perpetuates keratinocyte hyperproliferation, ultimately resulting in psoriatic plaque formation [45, 65]. Thus, CBDF may ameliorate psoriasis by intervening in this complex cellular and molecular network.

Our previous studies identified RHCG as a marker of abnormal KC differentiation and DC activation in psoriasis [13]. Recent findings from our laboratory have demonstrated that the overexpression of RHCG significantly upregulates the expression of KC differentiation markers, including S100 Calcium Binding Protein A family members (S100A) and Keratin 17 (KRT17), while concurrently suppressing Keratin 1 (KRT1) expression. Notably, this regulatory effect was accompanied by a marked increase in CXCL14 secretion from keratinocytes, which subsequently mediated DC activation through paracrine signaling pathways. These results collectively suggest that RHCG modulates KC phenotypic plasticity and immune microenvironment interactions via cytokine-mediated crosstalk [12]. In the current study, we observed a downregulation of RHCG following CBDF treatment. Given that RHCG may represent a critical node in KC–DC crosstalk, we examined the corresponding protein expression in CBDF-treated psoriatic mouse models. Immunofluorescence staining revealed that CBDF reduced the expression levels of both KRT17 and LAMP3. While these findings suggest a potential link between CBDF and RHCG-associated pathways, the role of RHCG as a downstream effector remains speculative and warrants further functional validation. Nonetheless, our results offer preliminary insights into possible molecular mechanisms by which CBDF may ameliorate psoriasis-related phenotypes.

We acknowledge several limitations of this study. First, large-scale, well-designed clinical trials are necessary to further substantiate the efficacy and safety of CBDF in the treatment of psoriasis. Second, more advanced experimental approaches will be employed to investigate the specific active compounds within CBDF and to elucidate the complex molecular regulatory networks underlying the synergistic actions of multiple constituents. Importantly, the use of TCM in psoriasis management carries potential risks of adverse effects. Certain herbal formulations, while offering therapeutic benefits, may induce skin irritation, gastrointestinal disturbances, or hepatic and renal dysfunction. Furthermore, interactions between TCM components and conventional medications may result in unforeseen pharmacological consequences. Therefore, careful monitoring and comprehensive evaluation of patients receiving TCM therapies are crucial to minimizing these risks. These considerations define the focus of our future research efforts.

## 5. Conclusion

This study investigated the anti-inflammatory mechanisms of CBDF in the treatment of psoriasis and demonstrated its therapeutic efficacy in a psoriatic mouse model. CBDF treatment led to a significant reduction in PASI scores, a marked decrease in epidermal thickness, and downregulation of key inflammatory markers including IL-17A, IL-23, and TNF-*α*. Mechanistically, our findings suggest that CBDF may modulate keratinocytes and DC dysfunction via RHCG-associated pathways. While RHCG is proposed as a potential downstream target of CBDF, further functional studies are required to confirm its causal role. Overall, this study provides new insights into the therapeutic action of CBDF and supports its potential utility in psoriasis management.

## Data Availability

We declare that all the data in this article are authentic, valid, and available for use on reasonable request.

## Supporting Information

Supporting Information Additional supporting information can be found online in the Supporting Information section. The details for herbal compositions in CBDF and all antibodies and reagents are provided.
