# Supplementary material for: Cooling Blood and Detoxicating Formula Treats Psoriasis Through RHCG-Related Mechanisms
Source: Int J Genomics. 2025 Jul 9;2025:5132158. doi: 10.1155/ijog/5132158 (PMC12267955; doi:10.1155/ijog/5132158)
Supplement: Supporting Information — Additional supporting information can be found online in the Supporting Information section. The details for herbal compositions in CBDF and all antibodies and reagents are provided. [file 5132158.f1.docx]

## Supplementary Table

### Table S1

**The list of herbal compositions in CBDF**

| Herbal name in Chinese | Latin name | Dosage |
| --- | --- | --- |
| Tufuling | Smilax glabra Roxb. | 30g |
| Shengdihuang | RehmanniaglutinosaLibosch. | 15g |
| Shenghuaihua | Black Locust Flower | 15g |
| Zicao | Radix arnebiae/Radix lithospermi | 10g |
| Caoheche | Paris polyphyllaSm | 9g |
| Chishaoyao | Paeoniae Radix Rubra | 10g |
| Jinyinhua | LoniceraeJaponicae Flos | 15g |
| Baimaogen | ImperataeRhizoma | 30g |
| Kushen | SophoraeFlavescentis Radix | 10g |
| Baixianpi | Dictamni Cortex | 10g |

### Table S2

**Detailed Information on Materials and Reagents Employed for Experimental Procedures**

| Antibodies and Reagents | Manufacturer, Country, Cat number, Lot number | Concentration |
| --- | --- | --- |
| RHCG Polyclonal antibodies | AiFang, China, AF06530, 20230302 | IF 1: 200 |
| Hematoxylin-Eosin staining Kit | Solarbio life sciences, China, G1120, 20231225 | - |
| 4',6-diamidino-2-phenylindole  (DAPI) | Beyotime Biotechnology, China, C1002, 091620210520 | - |
| Cytokeratin 17 Rabbit Polyclonal antibodies | Proteintech, USA,18502-1-AP00041309 | IF 1: 200 |
| LAMP3 Polyclonal antibodies | Thermo Fisher Scientific, USA,PA5-29566, YC3864379D | IF 1:200 |
| CoraLite488-conjugated Goat Anti-Rabbit IgG(H+L) | Proteintech, USA, SA00013-2, 205001014 | IF 1:300 |
| 4% Paraformaldehyde | Biosharp, China, BL539A,22329929 | - |
| Imiquimod Cream (IMQ) | iNova Pharmaceuticals,Singapore, H20160079,8825 |  |
| Mometasone Furoate Cream | Bayer Pharmaceuticals, Germany, H19991418, 240127 |  |
| Methotrexate | Shanghai Pharmaceuticals Holding Co., Ltd, China,H31020644, 197240603 |  |

## Supplementary Figure

### Figure S1


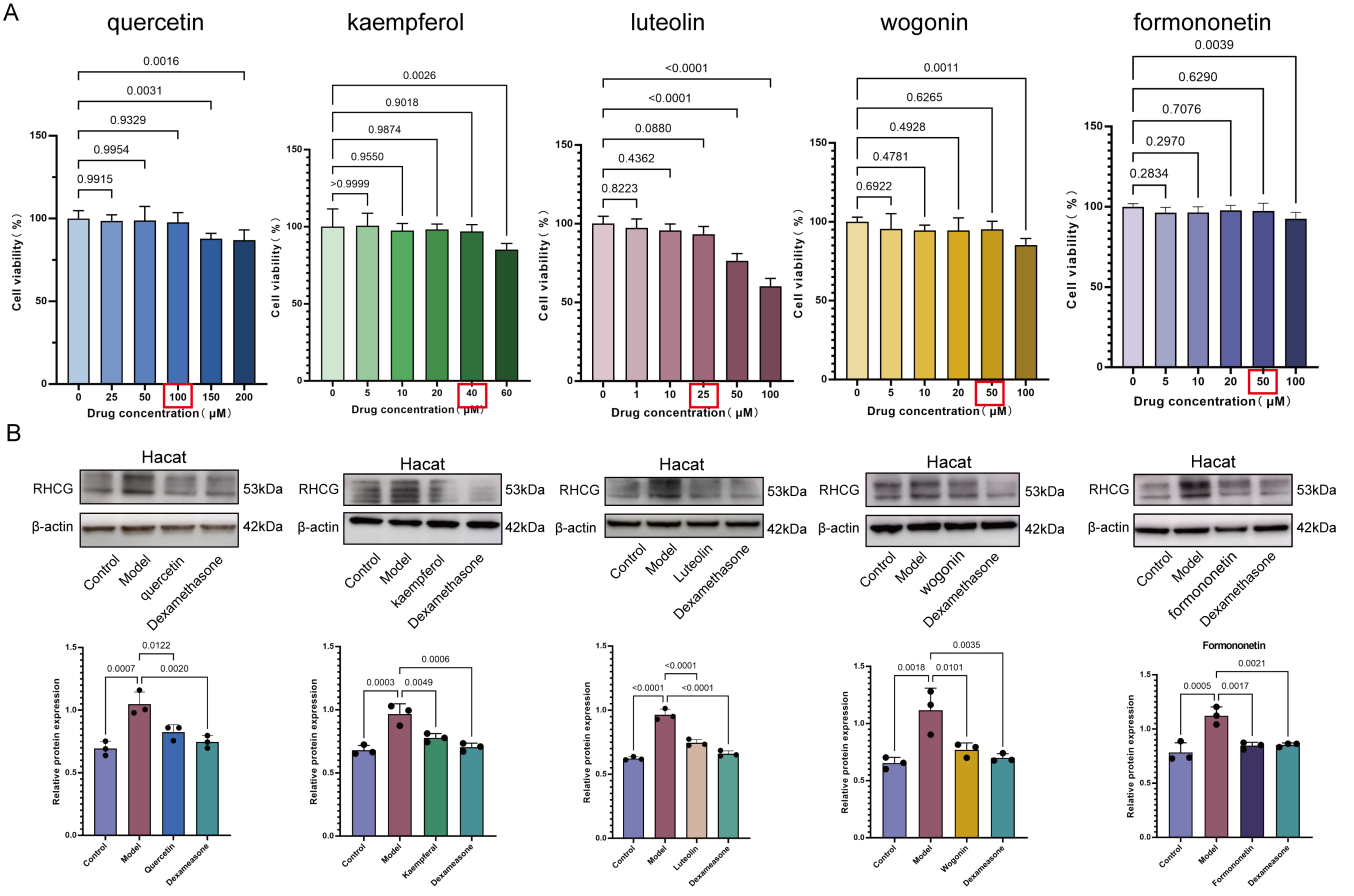


**Figure S1. Effects of Individual Compounds on Cell Viability and RHCG Expression in HaCaT Cells**

(A) Cell viabilityof HaCaT cells treated with quercetin, kaempferol, luteolin, wogonin, and formononetin at various concentrationsfor 24 hours, assessed by CCK-8 assay. Concentrations in the red box indicate the maximum non-toxic concentrations showing no significant impact on cell viability, which were used for subsequent Western blot experiments.

(B) Relative RHCG protein expression in HaCaT cells treated with the maximum non-toxic concentrations of each compound (as determined in panel A) compared to M5 model group and dexamethasone (positive control). Protein levels were analyzed by Western blot, with β-actin as a loading control.

Data are presented as mean ± SD(n=3), statistical significance was assessed using one-way ANOVA followed by Tukey’s post hoc test. P values are indicated for each comparison.
